# Chew and Spit (CHSP): a systematic review

**DOI:** 10.1186/s40337-016-0115-1

**Published:** 2016-08-22

**Authors:** Phillip Aouad, Phillipa Hay, Nerissa Soh, Stephen Touyz

**Affiliations:** 1 School of Medicine, University of Sydney, Sydney, Australia; 2 Centre for Health Research, School of Medicine, Western Sydney University, Penrith, Australia; 3 School of Psychology, University of Sydney, Sydney, Australia

**Keywords:** Chew and Spit, CHSP, Oral expulsion syndrome, Eating disorder, Anorexia, Bulimia, EDNOS, Abnormal eating, C/S, Chewing and spitting

## Abstract

**Background:**

This systematic review is an evaluation of the empirical literature relating to the disordered eating behaviour Chew and Spit (CHSP). Current theories postulate that CHSP is a symptom exhibited by individuals with recurrent binge eating and Bulimia Nervosa.

**Aims:**

The review aimed to identify and critically assess studies that have examined the distribution of CHSP behaviour, its relationship to eating disorders, its physical and psychosocial consequences and treatment.

**Methods:**

A systematic database search with broad inclusion criteria, dated to January 2016 was conducted. Data were extracted by two authors and papers appraised for quality using a modified Downs and Black Quality Index.

**Results:**

Nine studies met the inclusion criteria. All were of clinical samples and majority (*n* = 7) were of low quality. The pathological action of chewing food but not swallowing was reported more often in those with restrictive type eating disorders, such as Anorexia Nervosa, than binge eating type disorders. CHSP also was reported to be an indicator of overall severity of an eating disorder and to appear more often in younger individuals. No studies of treatment were found.

**Conclusions:**

Conclusions were limited due to the low quality and small numbers of studies based on clinical samples only. Further research is needed to address gaps in knowledge regarding the physiological, psychological, social, socioeconomic impact and treatment for those engaging in CHSP.

## Plain English Summary

Some people have a problematic weight control behaviour of chewing their food and spitting it out before swallowing, or Chew and Spit. We searched all the scientific papers that we could find on Chew and Spit for information but there were only nine studies and they could not be relied upon because of poor scientific quality. However, it seemed likely there was an association between Chew and Spit and eating disorders like anorexia nervosa. Studies of why people Chew and Spit, how it affects their health, and how to help them are needed.

## Background

Chew and Spit (CHSP) is the pathological behaviour of chewing a food, often of subjectively enjoyable quality as well as dense caloric content, and then spitting it out before swallowing as a means to avoid ingesting unwanted calories [1]. CHSP is an understudied weight control method possibly used as a binge eating compensatory behaviour employed by individuals with an eating disorder (ED).

The three most widely recognised ED diagnoses are Anorexia Nervosa (AN), typically characterised by behaviour of extreme calorie restriction, Bulimia Nervosa (BN) with its attendant behaviour of binging and purging (BP), and Eating Disorders Not Otherwise Specified (EDNOS) – including sub-threshold diagnoses of AN and BN – which has been revised into Unspecified Feeding and Eating Disorders (UFED) and Other Eating and Feeding Disorders (OSFED) in the DSM-5 [2–4]. Although the behaviour of CHSP is not found in current diagnostic schemes, it has been identified across the spectrum of EDs and was present in former diagnostic criteria such as the DSM-IV [5].

Individuals other than those with an ED may also engage in CHSP for a variety of reasons. For example, individuals undergoing bariatric surgery, those with diabetes, and athletes adhering to strict dietary guidelines may use CHSP to ‘taste’ food while adhering to their prescribed meal plans or eating requirements [6–8]. Many people also report disordered eating and ED behaviours pre and post bariatric surgery and it is likely that ED behaviours, including CHSP, are under reported and not detected during surgical assessments [7, 9–11]. However, the psychological and physiological effects of CHSP have yet to be delineated in any population.

The physiological process of preparing to receive food, called the cephalic response, is linked to metabolic changes in the body. Some studies involving modified sham feeding have focused on specific hormones, such as insulin, obestatin, and ghrelin as part of the cephalic response [11–17]. However, few studies involving sham feeding do so in the context of disordered eating [18–21]. Nor do sham-feeding studies focus on behaviour and psychological phenomenology, with specific studies into the influence of CHSP on metabolic responses being non-existent [19–25]. Empirical studies into the aetiology, psychological impacts, and physiological outcomes of CHSP would offer insight into an understanding of such processes in individuals with broader EDs, diabetes, or who are prone to post-bariatric-surgery dumping (the quick passage of food from the stomach to the small intestine) [18].

Hypothesised outcomes of CHSP may include weight gain due to accidentally ingesting calories, psychological and emotional distress (such as shame and guilt), and other physiological sequelae (such as damage to teeth, stomach ulcers, and hormonal imbalances) [6–9]. In addition to the possible psychological and physiological effects of CHSP, social consequences such as social isolation and financial strain (as seen in individuals with BN) may result, especially if an individual is prone to frequent CHSP ‘binge’ type episodes [26, 27].

This systematic review aims to examine existing evidence, identify, and critically examine studies that have investigated the distribution of CHSP behaviour, its relationship to EDs, and physical and psychosocial consequences. As the literature is sparse, the search and eligibility criteria were broad. The review also aims to identify gaps in the knowledge as, to date, little appears to be known about CHSP within and outside of EDs, including its prevalence, distribution, and putative harmful physical or psychological effects.

## Methods

### Search strategy and study eligibility criteria

**Fig. 1:**
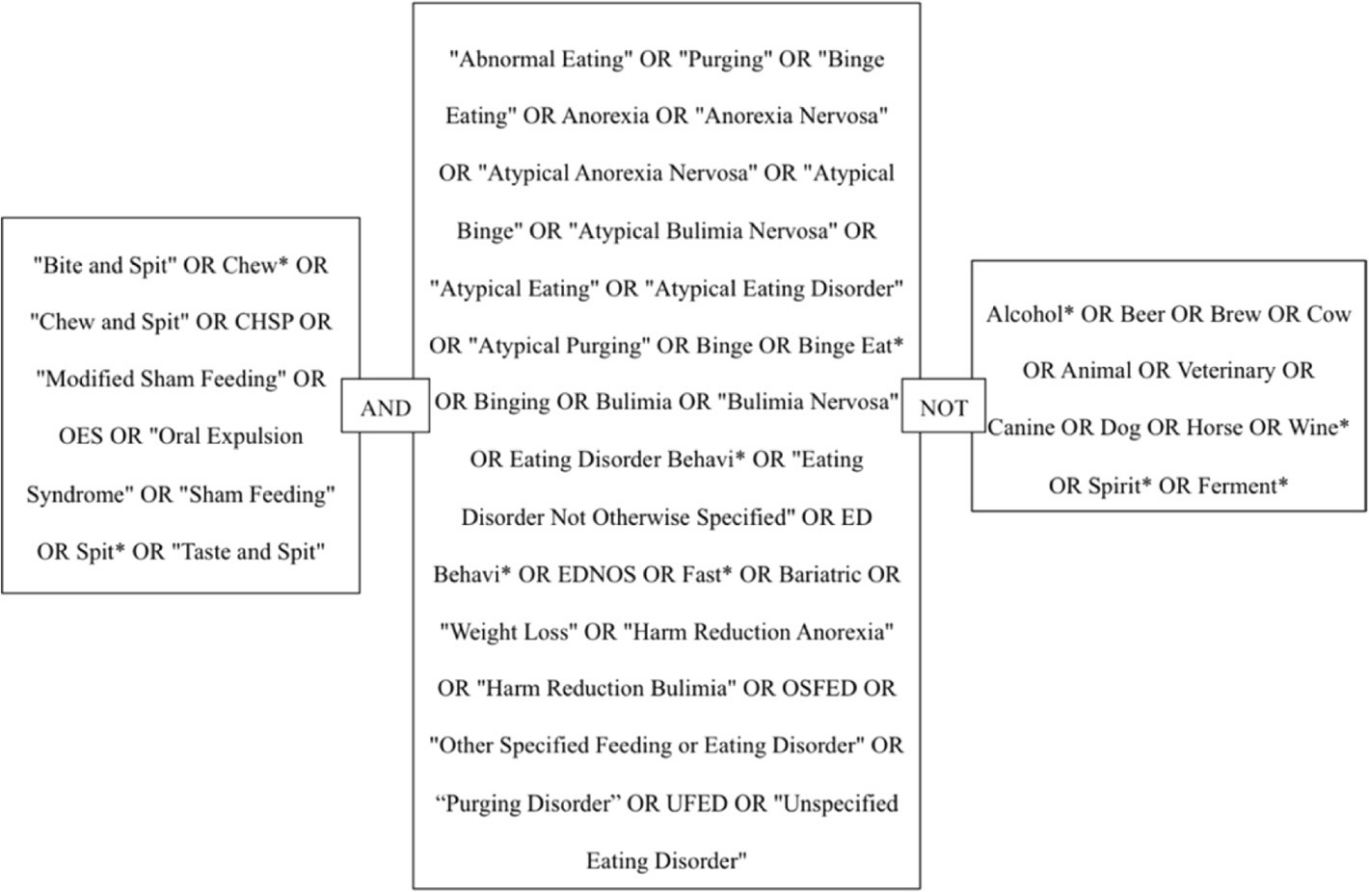
Search string used for database searching

The following electronic databases were searched: Medline, PubMed, Web of Science, the Cochrane Library, CINAHL, EMBASE, PsychInfo and Scopus using the search strings outlined in Fig. 1. Searches were conducted using key words and repeated using MeSH categories where applicable. References of included studies were manually screened by title. The full text for any potentially eligible studies was sourced and assessed for eligibility.

Inclusion criteria for studies are listed on Table 1. Only peer-reviewed studies were considered for inclusion to ensure data integrity and maintain quality. Studies of non-human participants were excluded as were those pertaining to rumination and pica, which are categorized in the DSM-5 as two distinct disorders separate to CHSP [2]. CHSP is distinctive from rumination, which is the regurgitation of stomach contents that is either chewed and re-swallowed, or spat out; and can occur involuntarily – often seen in those with severe purging subtype eating disorders as a motivated and habitual behaviour – or voluntarily [27]. On the other hand, pica involves the ingestion of non-food items [27]. The definition of CHSP thus was limited to the conscious chewing and spitting out of food only without regurgitation of swallowed food. As commonly seen in eating disorders, symptoms may overlap. However, studies were excluded if they did not focus on CHSP specifically or were related to regurgitation or chewing and spitting non-food items [27].

**Table 1:** Inclusion criteria for literature relating to ‘Chew and Spit’

Sample population	Humans only (ED or Non-ED)
Age group	Any
Condition	Participants must have a ‘lifetime history’ of CHSP (in conjunction with or without other ED behavior) and have exhibited the behavior prior to the study and not solely as part of another study with modified sham feeding. The main focus of the study must be centered on chewing and spitting out of food only, and not related to the regurgitation of swallowed food.
Study type & design	Any – including but not limited to RCTs, case studies and case series reports
Outcome measure	Assesses or explores some impact (physiological, social, or psychological) resulting from CHSP
Setting	No restriction
Date of study	All studies up to and including January 2016
Publication type & availability	Peer-reviewed and full-text only
Language	English only

Two authors (PA and NS) independently screened titles and abstracts of search results and full-text articles were retrieved of those studies that had potential to fit the inclusion criteria (Table 1). The two authors also assessed full-text articles to confirm that articles met to eligibility criteria. Disagreements were resolved through discussion, or referral to a third review author.

**Fig. 2:**
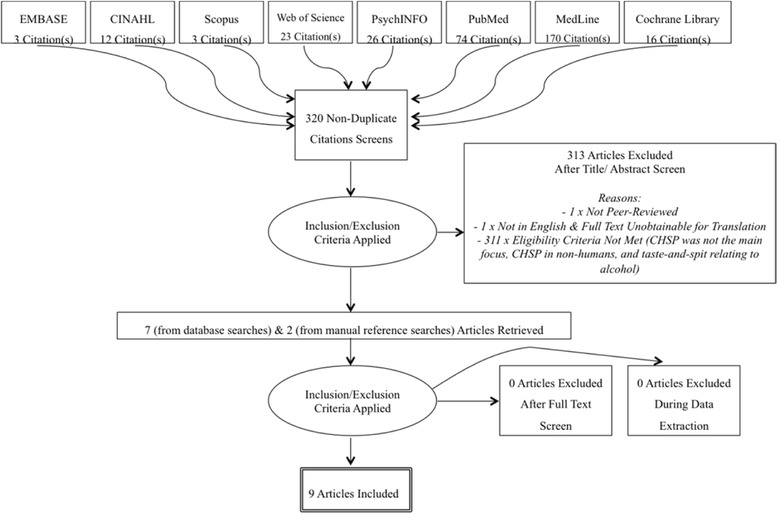
Flow diagram highlighting selection process of included articles [28]

A flow diagram in accord with PRISMA guidelines [28] of the number of identified records is depicted in Fig. 2.

### Data extraction

Data regarding CHSP symptomology, prevalence, psychological, social, or physiological impacts were extracted from the included studies. Data not directly related to CHSP were not included in the summary tables.

### Quality assessment

Study quality was assessed using a modified version of the Downs & Black Quality Index [29], as amended by Ferro and Speechley [30]. The quality index excluded items specifically related to randomised controlled trials (RCTs) such as those items assessing randomisation, dropouts, blinding, and intervention integrity: none of the included studies were RCTs. The amended version of the quality index had 15 items, as opposed to the original 27, and dichotomously scored various items as 0 (Unable to determine/No) and 1 (Yes). This resulted in subscales assessing reporting (7 items), external validity (3 items), internal validity (4 items), and study power (1 item). The maximum score was 15 with higher scores (>10) indicating higher methodological quality [30, 31].

## Results

Of the 320 studies identified, only nine met the eligibility criteria for inclusion in the systematic review – six cross-sectional studies, and three case studies. The Quality Index Scores were modest: of the nine studies, only two studies scored 10 while all other studies scored ≤ 9 (Table 2). No study reported response rates, adjusted for confounding or reported power size/sample calculation. All were clinical samples and none representative of the treatment majority. Given the heterogeneity across studies, data are presented qualitatively. Table 3 summarises the methods of included studies, while the main findings of each study are summarised below.

**Table 2:** Ferro and speechley quality index scores for included studies [30]

	Reporting							External validity			Internal validity (Bias and Confounding)				Power	
	Hypothesis or objectives described	Main outcomes described in introduction or Methods sections	Patient characteristics described	Main findings described	Estimates of the random variability	Probability values reported	Response rate described	Patients Asked to Participate Representative of Population	Patients Prepared to Participate Representative of Population	Staff, Places, and Facilities Representative of Treatment Majority	“Data Dredging” Made Clear	Statistical Tests Appropriate	Outcome Measures Valid and Reliable	Adjustment for Confounding	Sample Size or Power Calculation	Totals
Song et al. [34]	1	1	1	1	0	1	0	0	1	0	1	1	1	0	0	9
Guarda et al. [32]	1	1	1	1	0	1	0	0	1	0	1	1	1	0	0	9
De Zwaan [37]	1	0	0	1	0	0	0	0	0	0	0	0	0	0	0	2
McCutcheon & Nolan [39]	1	0	0	1	0	0	0	0	0	0	0	0	0	0	0	2
Makhzoumi et al. [36]	1	1	1	1	1	1	0	1	0	0	1	1	1	0	0	10
Kovacs et al. [38]	1	1	1	0	0	1	0	1	1	0	1	1	1	0	0	9
Durkin et al. [35]	1	1	1	1	1	1	0	1	0	0	1	1	1	0	0	10
Mitchell et al. [1]	0	1	1	1	0	0	0	0	0	0	0	0	1	0	0	4
Smith and Ross [33]	1	0	0	1	0	0	0	0	0	0	0	0	0	0	0	2

**Table 3:** Summary of included studies in this review of Chew and Spit behaviour

Study	Aims	Participants	Methodology	Assessment Tool/s	Setting	Inpatient (IP)/Outpatient (OP)/Partial outpatient (POP)
Song, Lee, Jung [28]	To investigate the relationship between CHSP and other ED related symptoms	359 Patients (Mean age = 23.2, SD = 6.6) diagnosed with EDs using DSM-IV-TR by a psychiatrist	Cross-Sectional Study. Results of ED patients who CHSP were compared to those who do not CHSP. ED symptoms compared included: EDs, Food Craving, Body Shape Dissatisfaction, Depression, Anxiety, and Obsessive Compulsive tendencies	Questionnaires including EDI-2, FCQ, BSQ, BDI, BAI, and MOCI	Mind & Mind ED Clinic presentations between 2010 and 2012 in Korea	Not Specified
Guarda, Coughlin, Cummings, Marinilli, Haug, Boucher and Heinberg [32]	To evaluate the prevalence and frequency of CHSP in trans-diagnostic ED patients	301 Patients (Mean age = 25, SD = 10) were diagnosed by trained interviewers using the structured clinical interview for DSM-IV	Cross-Sectional Study. Results of ED patients who CHSP were compared to those who do not CHSP. Questionnaires addressed demographics, ED symptoms and frequencies	Researcher developed questionnaire, EDI-2 and BDI.	Patients with consecutive admissions to a behavioural, inpatient, and partial hospitalization program for EDs	IP and POP
De Zwaan [37]	To present a novel case report on one patient	19-year-old female with a history of EDs (AN, 43 kg/15.6 kg/m2).	Case Study, 1 Patient	A case report	Psychotherapy treatment setting	OP
McCutcheon & Nolan [39]	To present a novel case report on two patients	Patient 1: 27 year old female Patient 2: 19 year old female college student	Case Report, 2 Patients	A case report of two subjects	Psychotherapy treatment setting	Not Specified
Makhzoumi, Guarda, Schreyer, Reinblatt, Redgrave, and Coughlin [36]	To characterize CHSP in a large sample of ED inpatients treated in a hospital-based behavioural speciality program. To investigate associations between regular CHSP and personality dimensions, ED and depression symptomology, and short-term clinical outcome variables. To examine CHSP including the amount of food typically consumed and frequency of Loss of Control (LOC) associated with CHSP behaviour.	324 Patients (Mean age = 29, SD = 12.4) were diagnosed by trained interviewers using the structured clinical interview for DSM-IV	Cross-Sectional Study. Results of ED patients who CHSP were compared to those who do not CHSP. Questionnaires addressed demographics, ED symptoms and frequencies	Frequency and overall number of nine types of current and lifetime ED behaviours were assessed using a researcher developed questionnaire, BDI, EDI-2, NEO-FFI.	Patients with consecutive admissions to an integrated inpatient partial hospital treatment program for EDs who agreed to participate in an outcome study	IP and POP
Kovacs, Mahon, and Palmer [38]	To study the prevalence and association of CHSP in a series of patients with AN, BN, and EDNOS	710 adult patients (Mean age not specified) were diagnosed according to the criteria outlined in the DSM-III-R	Cross-Sectional Study. ED patients who CHSP were compared between ED subtypes (AN, BN, and EDNOS) and those who did not engage in CHSP	Clinical Eating Disorder Rating Instrument (CEDRI), defined binging (DSM-III-R definition), subjective overeating, and subjective distortion of body image.	Inpatient ED Service of the Leicester General Hospital between 1991 and 1998	IP
Durkin, Swanson, Crow, Mitchell, Peterson, and Crosby [35]	To promote cohesion between existing CHSP literature (CHSP is trans-diagnostic) from an out-patient perspective	972 outpatients (Mean age = 24.6, IQR = 20.66–31.10)	Cross-Sectional Study. Patients were classified as having current CHSP behaviour or having had CHSP (at any frequency) during their lifetime.	EDQ. CHSP behaviour was assessed was determined by using 2 general EDQ items.	Patients evaluated at the Outpatient ED Clinic at the University of Minnesota between 1985 and 1996	OP
Mitchell, Pyle, Hatsukami, and Eckert [1]	A presentation of information about CHSP in BN patient engaging in the behaviour at high frequency, including information about their associated ED symptoms, treatment histories, and related psychopathology	Patients (Mean age = 23.9 y, SD = 5.3) who presented at an ED clinic prior to the commencement of the study.	Retrospective analysis. 25 patient files were retrospectively examined for indications of CHSP	Files of patients were retrospectively evaluated and diagnosed based on the information present in the files that corresponds to the DSM-III BN criteria and who engaged in CHSP	Files of patients evaluated at the ED Clinic at the University of Minnesota over three years prior to this 1987 study.	Not Specified
Smith and Ross [33]	To present a novel case report on one patient	28-year-old Caucasian obese female with no previous history of ED behaviour, but with a history of treatment for bipolar disorder.	Case Study, one Patient	A case report	Psychotherapy treatment setting/physician directed PSMF	OP

### Main findings

The nine studies included indicate that CHSP has been investigated in predominantly ED samples [1, 32–39]. The results below indicate a number of similarities between individuals with an ED who engage in CHSP.

As shown in Table 3, one cross-sectional study by Song et al. (*N* = 359) [34], found that 24.5 % of participants with EDs and with CHSP had more pathological eating behaviours, higher levels of food craving, more concerns regarding body shape, and higher levels of mood and anxiety related symptoms [34]. CHSP was found to be trans-diagnostic (occurring in AN, BN, and EDNOS diagnosed individuals), associated with more pathological compensatory behaviours, and correlated with greater ED severity [27].

Similarly, a cross-sectional study by Guarda et al. [32] (*N* = 310) reported that individuals with EDs engaging in CHSP (CHSP+) were be younger (mean age 22.6 years, SD 7.2) than those who did not (CHSP-; mean age 25.6 years, SD 10.5). However, length of illness was not associated with this finding. Compensatory behaviour was more prevalent in the CHSP+ group. CHSP was reported to be trans-diagnostic and was more strongly correlated with restrictive behaviours than with elevated purge behaviour (CHSP+/B-). The majority (76 %) of CHSP+/B+ had a diagnosis of BN and the majority of those who binged and engaged in CHSP (87 %) also engaged in purging. There was no significant difference in in psychometric measures (i.e., BDI, EDI-2, length of illness, and dieting history) between participants with CHSP+/B+ and CHSP+/B-. The results of this study indicated CHSP was positively associated with ED severity [32].

Makhzoumi et al. [36] reported that, in a cross-sectional study, 34 % of respondents (*N* = 324) with CHSP had binge-like quantities of food (≥ or = 1000 calories) in their lifetime, and 18 % reported this in the 8 weeks before admission to an ED clinic. Nine out of the 10 individuals engaging in CHSP at the time of the study reported a subjective sense of loss of control (LOC) at some point in their lifetime, with a majority of CHSP+ (70 %) reporting LOC even when they CHSP non-binge quantities. CHSP groups did not differ in demographic features, clinical indices, or CHSP frequency across ED diagnostic groups. However individuals who CHSP, were more likely to have a purging, as opposed to restricting, diagnosis. Nonetheless, after controlling for behavioural subtype, individuals with CHSP engaged more frequently in restrictive eating behaviours, diet pill, laxative abuse, and over-exercise [36]. Additionally, CHSP+ participants engaged more frequently in binge eating and appeared to engage in a wider array of ED behaviours than CHSP- participants. Finally, CHSP participants had also exhibited greater drive for thinness, body dissatisfaction, and higher depressive symptomology and neuroticism (even after controlling for behavioural subtype), and were more likely to endorse suicide ideation. Makhzoumi et al. concluded that CHSP should be assessed in all ED individuals, as neuroticism is a risk factor for ED behaviour and is positively correlated with ED symptoms [36].

In a cross-sectional study, Kovacs et al. [38] found that people with EDNOS (*N* = 344) and AN (*N* = 124) who reported CHSP showed more severe eating behaviour pathology compared to participants with BN and CHSP (*N* = 242). In contrast, participants with BN and CHSP reported a greater distortion of body image compared to their AN and EDNOS counterparts. CHSP was trans-diagnostic and positively correlated with laxative abuse in individuals with AN. The individuals with EDNOS who engaged in CHSP appeared to exhibit traits concordant with AN. Subjective overeating appeared to be a positive predictor of CHSP in the AN group.

Results of a cross-sectional study by Durkin et al. (*N* = 972) [35] indicated an overall positive association between being diagnosed with an ED and lifetime CHSP, with CHSP frequencies ranging from less than or equal to once a month to several times a day. The majority (67.9 %) of those who engaged in CHSP at some point in their lifetimes generally were still currently engaging in the behaviour at the time of study. The results also indicated that participants who have had a lifetime history of CHSP were more likely to have been diagnosed with AN or BN as opposed to EDNOS. However, the authors did not indicate if CHSP was a risk factor for developing a clinical ED in the future [35]. Findings of the study indicated that CHSP was not trans-diagnostically present –unlike in the other cross-sectional studies - but was more commonly associated with those with an AN or BN diagnosis as opposed to EDNOS. Younger participants were more likely than older participants to engage in CHSP. Furthermore, it was demonstrated that CHSP was associated with both past and present restrictive behaviour and CHSP is usually used as a short-term compensatory behaviour for some individuals with EDs [35].

In a cross-sectional study Mitchell et al. [1] (*N* = 25) found 68 % of the 25 participants reported CHSP with 8 % indicating having had CHSP at a minimum frequency of several times a week at some point during their illness. The authors posited that CHSP is used as a substitute for binging and purging or other bulimic behavioural patterns. In this study CHSP occurred at a low frequency in women with BN [1]. When comparing individuals with BN who had low frequency CHSP to those with high frequency CHSP, there was no significant difference between these types of participants and a control group [1].

Of the included studies, three were case reports (De Zwaan [37], McCutcheon and Nolan [39], and Smith and Ross [33]) outlining participants’ histories, both in the context of CHSP and wider ED behaviour. In total, the case studies report on four females aged between 19 and 28. Similar themes between the reports highlight that CHSP was used as a weight-control method and was often associated with negative emotions such as self-disgust, remorse, and shame, but may have been be less distressing than binging and purging [33, 37, 39]. Additionally, because of CHSP, individuals appeared to have concerns over social, financial, and familial issues [33, 37, 39]. Only one study, by Smith and Ross [33] offered possible explanations for the CHSP behaviour, including avoiding feeling deprived, addiction transference, a stress response, or a deficiency in trace minerals or vitamins.

## Discussion

This systematic review identified nine studies that met the eligibility criteria [1, 32–39]. Grey literature, clinical but non-academic sources, and other sources that did not meet the eligibility criteria were surveyed to generate plausible hypotheses and mechanisms for describing pathways and outcomes of CHSP. However, the prevalence of CHSP remains unclear due to the paucity of peer-reviewed academic literature on CHSP and the available studies being case studies or cross-sectional studies with small sample sizes that are mostly comprised of adult female participants. The psychological, social, or physiological precursors or outcomes of the behaviour also remain unclear. Moreover, the Ferro and Speechley [29] index scores raised concerns about the quality of studies, with no single study scoring above ten on a fifteen-point scale. Further to this, none of the studies made comment on probable resulting referral bias or the differences in comorbidity dependant on inpatient or outpatient setting and clinical specialties (see Table 3 for included study settings). Such referral bias could have given rise to higher numbers of participants with specific eating disorders than would sampling CHSP across the ED spectrum. The quality of eligible studies was of concern as no RCTs were conducted to investigate responses to various treatment options for the disordered behaviour of CHSP. Overall, the included studies did not provide deep insight into the wider prevalence and consequences of CHSP.

The included studies reported a wide range of frequency of CHSP in ED samples ranging from 22 to 100 %. With the exception of diuretic and laxative misuse, the disordered behaviours of binge eating and dietary restriction were very common (ranging over 70 %) in people with lifetime or current CHSP [32, 34, 36, 38]. Both Durkin et al. [35] and Guarda et al. [32] reported that CHSP appears to be more common in younger individuals with eating disorders.

All included studies except for Smith and Ross [33] focused on the behaviour of CHSP exclusively in ED individuals. Smith and Ross [33] presented a case study on CHSP symptomology in a patient who was not diagnosed with an ED but who turned spontaneously to the behaviour during a period of extreme calorie and nutrient deprivation akin to that experienced during disordered eating patterns.

Further to this, CHSP may be an indicator of ED behavioural severity [32]. As ED illness severity increases, individuals may experiment with more ED behaviours such as CHSP. CHSP may serve as a way to taste ‘forbidden’ or feared food, and it is possible that CHSP ‘binging’ may develop as a substitute for regular binging and purging even if the associated risks with the behaviour appear elevated [32, 34, 36, 39].

While the participants in the Mitchell et al., [1] study were not studied systematically relative to treatment they received, a review of their charts indicated that most were able to participate in an outpatient program for BN and had apparent success (however no follow-up study was conducted). It appeared that those that engaged in CHSP at high frequencies may eventually cease or decrease the behaviour with most not using CHSP as a substitute for purging (laxative or vomiting) but may rather alternate between the two behaviours [1].

Prior to the DSM-5 [2, 5], CHSP was included under the EDNOS classification. However, in the DSM-5 CHSP behaviour has been removed from the definition of OSFED and UFED (formerly EDNOS) with no explanation or transfer to another ED diagnostic category. One possible reason for this is that CHSP crosses ED diagnostic boundaries. Studies included in this systematic review indicate that CHSP is likely to be a trans-diagnostic behaviour [34, 36, 38]. Therefore, it is the recommendation of the authors of the present systematic review that clinicians consider inquiring about CHSP in all people presenting with an ED (or disordered eating).

This review identified a number of limitations in the current research centred on CHSP, all of which appeared to be of modest quality and, one-third of what little literature was found came from case studies. Only one case study involved a person without an ED and few studies undertook an in-depth analysis of the physical, social, or psychological implications of CHSP, with no studies deeply investigating physical or social impacts. None of the studies investigated CHSP in children or as a precursor to EDs later in life. We found no longitudinal studies and men and children with CHSP were severely under-represented in study samples. It is likely that the bias towards female participants was due to the study samples being based in ED clinics and it is known men with EDs are less likely to access treatment services than women [40]. However, it is also indicative of the relative neglect of men in ED research [40–42].

Longitudinal studies are needed to determine the prolonged effects of CHSP and the impacts on physiological, psychological, and social well-being. Such studies would provide greater insight treatment design for those that engage in CHSP but may not meet full diagnostic ED criteria.

A limitation to this review is that it sourced only English-language studies. One study in Japanese was present in the original literature search and there may have been others if non-English language databases had been searched. This would potentially have increased the number of studies and study participants of non-Caucasian descent.

Another limitation of this systematic review was its inability to source empirical studies on CHSP practices in populations without EDs but with specific dietary requirements, such as individuals with diabetes, athletes, bodybuilders, and bariatric patients. As bariatric surgery is becoming more commonplace in the treatment of morbid obesity, it would be relevant to ascertain how common CHSP is in this population [43]. The impact of CHSP on weight loss, as well as possible psychological and medically adverse complications in these groups, is at this time uncertain. The focus of published studies on CHSP and its association with EDs rather than in individuals who exhibit CHSP alone may result in some ‘at-risk’ people not being clinically identified as engaging in CHSP.

Further and higher quality research into CHSP is needed to provide greater understanding across several areas including: gaining insight into CHSP treatment, along with other more common ED types; bringing relief and insight for those engaging in CHSP; assisting clinicians who seek to treat individuals overcome CHSP behaviours; and providing clinicians with clear guidance for screening and treatment of CHSP [44]. Studies should also investigate the precursors and outcomes of CHSP not only in individuals with EDs but also in undiagnosed individuals who engage in CHSP and those who may be at risk of beginning the behaviour.

## Conclusion

This systematic review of CHSP literature revealed no consensus on the ED typology most closely associated with CHSP. The small number of poor quality studies published demonstrates that CHSP is an understudied topic.

Higher quality studies, including qualitative, quantitative, mixed-methods, and longitudinal studies, are required to add depth to clinical, physiological, psychological, and socioeconomic understandings of CHSP and its treatment. Such studies would assist in determining if there is a common psychological link between individuals with CHSP behaviour with and without an ED.
